# Etiology, Risk Factors, and Outcomes of Neonatal Candidiasis: Findings From a Retrospective Cohort Study

**DOI:** 10.7759/cureus.76773

**Published:** 2025-01-01

**Authors:** Ismail M Alwadani, Mohammed H Hakami

**Affiliations:** 1 Neonatology, Johns Hopkins Aramco Healthcare, Dhahran, SAU; 2 Neonatology, King Fahad Central Hospital, Jazan, SAU

**Keywords:** clinical course, etiology, neonatal candidiasis, risk factors, saudi arabia

## Abstract

Background

*Candida* is a common cause of invasive fungal infection, which increases the risk of morbidity and mortality in neonates. The prevalence of neonatal candidemia has been reported in some regions; however, there is a dearth of studies on the prevalence, risk factors, and outcomes of neonatal candidemia in Arab countries. Therefore, we conducted this study to determine the prevalence, etiology, risk factors, and adverse outcomes of neonatal candidiasis in Saudi Arabian neonatal populations.

Methods

This was a retrospective cohort study of neonates admitted to the intensive neonatal care unit of our institution between January 2010 and December 2020. A standard questionnaire was used to collect data on sociodemographic and clinical factors, clinical course, and survival. Multivariate logistic regression analysis was performed to determine the adjusted effects of the risk factors for neonatal candidiasis, as well as its clinical course and outcomes.

Results

A total of 80 neonates were analyzed in this study. Approximately 30% of the neonates had positive *Candida* cultures, with *Candida albicans* being the predominant species (72.4%). Regarding the clinical course, 41.4% of the neonates were stable, whereas 58.9% died during treatment. Neonatal birth weight (odds ratio, OR = 0.16; 95% confidence interval, CI: 0.023-0.89), length of hospital stay before candidiasis diagnosis (OR = 1.16; 95% CI: 1.03-1.31), and total length of hospital stay (OR = 0.93; 95% CI: 0.88-0.97) were significant risk factors for mortality. Total length of hospital stay was the only demographic risk factor associated with the clinical course of neonatal candidiasis (OR = 0.97; 95% CI: 0.94-1.00); however, the association was marginally significant.

Conclusion

The results of this study suggest that the prevalence of neonatal candidiasis in Saudi Arabia is high, and that greater attention needs to be paid to low-birth-weight babies with a prolonged hospital stay. The results of this study can facilitate the development of a framework or treatment protocol specifically designed for neonates susceptible to candidiasis.

## Introduction

Neonates are susceptible to fungal infections, mainly by the *Candida* species, shortly after birth [1]. *Candida* is a common cause of invasive fungal infections in neonatal intensive care units and is the third most commonly isolated microorganism in neonatal sepsis [2]. *Candida* causes approximately 2%-4% of infections in early neonatal sepsis and 10%-12% of infections in late neonatal sepsis [3,4]. In a five-year prospective study conducted in Kuwait, 4.6% of the early neonatal sepsis cases and approximately 11% of the late neonatal sepsis cases were caused by *Candida* species [5]. Neonatal *Candida* colonization is most common in the respiratory and gastrointestinal tracts during the initial 14 days of life, most likely due to vertical transmission [6].

The etiology of neonatal candidiasis is multifactorial; however, it is commonly associated with gastrointestinal tract immaturity, premature birth, improper development of the epidermis, and increased susceptibility to infection due to weak physical and immunological barriers [3]. Furthermore, hospital-related factors, such as the prolonged use of broad-spectrum antibiotics, parenteral nutrition, indwelling central lines, and endotracheal intubation, increase the risk of neonatal candidiasis [7,8]. Neonatal candidiasis, regardless of its etiology, increases the risk of neonatal morbidity and mortality, which is elevated in extremely low-birth-weight newborns [9,10]. The mortality rate of neonatal candidiasis is reported to range from 15% to 60%. A study conducted in Kuwait indicated that the mortality rate of neonatal candidiasis is approximately 22%, which is higher than that of any bacterial or other type of infection [5].

Early diagnosis of neonatal candidiasis is challenging for pediatricians and microbiologists due to the lack of specific signs and symptoms associated with the infection, and the unavailability of sensitive and specific diagnostic laboratory tests for neonatal candidiasis. The prevalence of neonatal candidiasis in some regions has been reported in previous studies. However, only a few studies on the prevalence, risk factors, and mortality outcomes of neonatal candidiasis in Saudi Arabia have been conducted. Therefore, we conducted this study to determine the prevalence, etiology, risk factors, and adverse outcomes of neonatal candidiasis in a single center in Saudi Arabia. The findings of the study will advance the understanding of the etiology of neonatal candidiasis and help neonatologists develop effective preventive and therapeutic measures for timely treatment and management of neonatal candidiasis, thereby reducing neonatal morbidity and mortality rates attributed to the infection.

## Materials and methods

Study design, setting, and eligibility criteria

This was a retrospective cohort study of neonates admitted to the intensive neonatal care unit of our institution between January 2010 and December 2020. All the neonates included in the study had positive *Candida* cultures. We used existing data extracted from the available medical records of the neonates; thus, informed consent from their parents was not required. We included all neonates with candidiasis admitted between 2010 and 2020. We followed the standard treatment protocol for the admitted neonates and analyzed their clinical outcomes using standard questionnaires.

Data collection

Data on important sociodemographic variables, such as gestational age at diagnosis (in weeks), sex (female/male), birth weight (low birth weight: <2500 g, very low birth weight: <1500 g, and extremely low birth weight: <1000 g), nationality, maternal antenatal corticosteroid use, mode of delivery, and age at the time of admission, were collected from the medical records of the neonates using standard forms. We also collected data on the status of fungal infection, such as type of culture, *Candida* growth on cultures, *Candida* species, *Candida*-positive blood culture, and whether the neonate received antifungal prophylaxis. In addition, data on potential risk factors, such as total parenteral nutrition, presence of a central line, use of antibiotics and its duration, use of anti-reflux medications, overall clinical course, respiratory support, feeding status of the child, and history of any surgery after birth, were collected. Data on adverse outcomes, such as length of hospital stay and neonatal survival or mortality, were also collected.

Statistical methods

Variables related to the characteristics of the study population are presented as frequencies and proportions, where appropriate. Categorical and dichotomous variables, such as sex, nationality, maternal antenatal corticosteroid use, and mode of delivery, are presented as frequencies and proportions. Non-skewed continuous variables (neonatal age and birth weight) are presented as mean and standard deviation (SD). Non-normally distributed data are presented as median and interquartile range. The Chi-square test or Fisher’s exact test was used to analyze the association between different categorical variables and adverse outcomes, such as mortality. An independent t-test or the Mann-Whitney U test was used to analyze differences in continuous variables. Logistic regression analysis was performed to determine the effect of each significant predictor on clinical outcomes and clinical course. Multivariate logistic regression analysis was performed to determine the adjusted effects of the risk factors for neonatal candidemia and its clinical course and outcomes. The results of the regression analysis are presented as adjusted odds ratios (ORs) with 95% confidence intervals (CIs). All the study data were analyzed using IBM SPSS Statistics for Windows, Version 25 (Released 2017; IBM Corp., Armonk, NY, USA). Statistical significance was set at a p-value of <0.05.

## Results

A total of 5,600 neonates were admitted to the neonatal intensive care unit of our institution between January 2010 and December 2020. Of these, 80 tested positive for *Candida* infection and were eligible for inclusion in this study. The sociodemographic and clinical characteristics of the neonates included in this study are shown in Table 1. The mean gestational age at diagnosis was 33.4 weeks (SD, 5.2 weeks), and their mean birth weight was 1.840 g (SD, 0.95 g). Approximately 65.5% of the neonates were male, and 34.5% were female. Regarding other characteristics, 74.1% of the neonates were Saudi; the mothers of only 18.8% of the neonates received a full course of antenatal corticosteroids, and 81.0% were delivered vaginally.

**Table 1:** Sociodemographic data and obstetric history of the included mothers and the fungal infection statuses of the neonates (n = 58) Variables related to the characteristics of the study population are presented as frequencies and proportions where appropriate. Non-skewed continuous variables (neonatal age and birth weight) are presented as mean and standard deviation (SD). PROM: prolonged rupture of membrane; ETT: endotracheal tube

Sociodemographic data on obstetric history of included mothers		Frequency		Percentage
Sex	Male	38		65.5%
	Female	20		34.5%
	Ambiguous	0		0.0%
Nationality	Non-Saudi	15		25.9%
	Saudi	43		74.1%
Maternal antenatal corticosteroid course	Full course	9		18.8%
	Single dose	14		29.2%
	No	25		52.1%
PROM (Prolonged rupture of membrane)	Yes	15		25.9%
	No	43		74.1%
Mode of delivery	Vaginal	47		81.0%
	Assisted	0		0.0%
	Cesarean section	11		19.0%
Gestational age at diagnosis (weeks) (mean ± SD)		33.4		5.2
Birth weight (kg) (mean ± SD)		1.840		0.954
Fungal infection statuses of neonates admitted to the hospital				
		Frequency	Percentage	
Type of culture	Blood	46	79.3%	
	ETT	3	5.2%	
	Urine	2	3.4%	
	Wound swab	3	5.2%	
*Candida* growth in cultures	Yes	17	29.8%	
	No	40	70.2%	
*Candida* species	Albicans	42	72.4%	
	Other species	16	27.6%	
*Candida*-positive blood culture	None	2	3.4%	
	Once	31	53.4%	
	Several	25	43.1%	
Sensitivity	Not mentioned	13	22.4%	
	Sensitive to all antifungals	45	77.6%	
Disseminated candidiasis	Yes	7	12.1%	
	No	51	87.9%	
Received antifungal prophylaxis	Yes	3	5.2%	
	No	55	94.8%	
Received postnatal corticosteroid	Yes	27	47.4%	
	No	30	52.6%	

The fungal infection statuses of the neonates are outlined in Table 1. Approximately 30% had positive *Candida* cultures, and the most common *Candida* species was *Candida albicans*. Regarding antifungal resistance, 77.6% of the infections were sensitive to all antifungal agents. Approximately 12.1% of the neonates had disseminated candidiasis; 5.2% received antifungal prophylaxis, and 47.4% received postnatal corticosteroids.

Data on the clinical features and management of fungal infections in neonates are presented in Table 2. The mean length of hospital stay before the diagnosis of candidiasis was 25 days (SD, 26 days), whereas the mean duration of the antibiotic course was 19 days (SD, 14 days). Approximately half of the neonates were administered antibiotics based on culture findings and sensitivity, and 100% of the neonates were placed on antibiotics for at least three days. Approximately 86.0% of the neonates had a central line, 89.7% were placed on total parenteral nutrition for more than a week, and 91.4% were placed on NPO (nil per os) status for more than seven days. Approximately 85% of the neonates were placed on some form of respiratory support, approximately one-third underwent surgery, and 8.6% received anti-reflux medications (Table 2).

**Table 2:** Clinical features and management of fungal infection among neonates (n = 58) Length of stay and duration of antibiotics are presented as mean and standard deviation (SD). NPO: nil per os; CPAP: continuous positive airway pressure; HFOV: high-frequency oscillatory ventilation

		Frequency	Percentage
Length of hospital stay before candidiasis diagnosis (days) (mean ± SD)		25	26
Duration of antibiotic use (days) (mean ± SD)		19	14
Use of antibiotics before candidiasis diagnosis	Based on culture	29	50.0%
	Empirical	29	50.0%
Antibiotics course prior to candidiasis diagnosis	≥3 days	58	100%
	<3 days	0	0%
Presence of central line	≥14 days	49	86.0%
	<14 days	8	14.0%
Total parenteral nutrition	≥7 days	52	89.7%
	<7 days	6	10.3%
NPO	≥7 days	53	91.4%
	<7 days	5	8.6%
Patient experienced necrotizing enterocolitis	Yes	17	29.3%
	No	41	70.7%
Underwent surgery	Yes	21	36.2%
	No	37	63.8%
Anti-reflux medications	Yes	5	8.6%
	No	53	91.4%
Overall clinical course	Stable	24	41.4%
	Unstable	34	58.6%
Respiratory support	No	9	15.5%
	Nasal cannula	1	1.7%
	CPAP	7	12.1%
	Conventional ventilation	24	41.4%
	HFOV	17	29.3%
Outcome	Cured	23	41.1%
	Died	33	58.9%
Total length of hospital stay (mean ± SD)		46	40

Results of the multivariate logistic regression analysis

The multivariate logistic regression model showing the clinical risk factors associated with the clinical course and outcomes of neonatal candidiasis is shown in Table 3. No risk factor was significantly associated with the clinical course and outcomes of neonatal candidiasis (i.e., no risk factor had a p-value of <0.05). However, neonates with positive *Candida* blood cultures were 1.2 times more likely to die (OR = 1.2; 95% CI: 0.30-4.77) than those with a negative blood culture; however, this result was not statistically significant. In addition, neonates with disseminated candidiasis were 1.21 times more likely to die than neonates without disseminated candidiasis (OR = 1.21; 95% CI: 0.107-13.54); however, this result was also statistically non-significant. The multivariate analysis did not reveal any statistically significant risk factors for mortality among neonates with candidiasis (Table 3). Similarly, the multivariate analysis did not reveal any statistically significant risk factors for the clinical course of neonatal candidiasis (Table 3). Neonates with a positive *Candida* blood culture were 1.91 times more likely to be unstable (OR = 1.91; 95% CI: 0.71-5.15) than those with a negative blood culture; however, the result was statistically non-significant. Neonates who were placed on antibiotics before the diagnosis of candidiasis had a 16.0% increased risk of having an unstable clinical course (OR = 1.16; 95% CI: 0.39-3.36); however, this result was statistically non-significant as well.

**Table 3:** Multivariate logistic regression model showing the clinical risk factors associated with the clinical outcome and course of neonatal candidiasis (n = 58) The Chi-square test or Fisher’s exact test was used to analyze the association between different categorical variables and adverse outcomes. A p-value of <0.05 is considered as statistically significant. OR: Odds ratio; 95% CI: 95% confidence interval

Risk factors associated with clinical outcomes								
Risk factors	Correlation coefficient	SE		Wald	p-value	OR	95% CI	
*Candida*-positive blood culture	0.18	0.704		0.067	0.79	1.20	0.30	4.771
*Candida* growth	-1.01	1.03		0.95	0.33	0.36	0.04	2.78
Antibiotics before candidiasis diagnosis	-0.18	0.69		0.074	0.78	0.82	0.21	3.20
Presence of central line	-0.71	1.23		0.332	0.56	0.49	0.04	5.49
Total parenteral nutrition	-0.52	1.54		0.116	0.73	0.59	0.03	12.15
Disseminated candidiasis	0.19	1.23		0.023	0.88	1.20	0.11	13.54
Underwent surgery	-1.13	0.78		2.10	0.15	0.32	0.07	1.48
Risk factors associated with clinical course								
*Candida*-positive blood culture	0.64		0.51	1.62	0.20	1.91	0.71	5.15
*Candida* growth	0.11		0.60	0.03	0.85	1.11	0.34	3.62
Antibiotics before candidiasis diagnosis	0.14		0.54	0.07	0.79	1.16	0.39	3.36
Presence of central line	-0.98		0.79	1.56	0.21	0.37	0.08	1.75
Total parenteral nutrition	-2.18		1.13	3.7	0.05	0.11	0.01	1.04
Disseminated candidiasis	-0.37		0.91	0.16	0.68	0.69	0.11	4.12
Underwent surgery	-0.74		0.59	1.55	0.21	0.48	0.10	1.52
Anti-reflux medications	-0.79		1.18	0.440	0.51	0.45	0.04	4.66

The multivariate logistic regression model showing the demographic risk factors associated with the clinical course and outcomes of neonatal candidiasis is shown in Table 4. A few factors were significantly associated with the clinical course and outcomes of neonatal candidiasis (i.e., factors with a p-value of <0.05). Neonatal birth weight (OR = 0.16; 95% CI: 0.023-0.89), length of hospital stay before candidiasis diagnosis (OR = 1.16; 95% CI: 1.03-1.31), and total length of hospital stay (OR = 0.93; 95% CI: 0.88-0.97) were significantly associated with mortality in the neonates. Specifically, the odds of mortality decreased by 84% and increased by 16% with a one-unit increase in birth weight and a one-day increase in length of hospital stay, respectively. Total length of hospital stay was the only demographic risk factor associated with the clinical course of neonatal candidiasis (OR = 0.97; 95% CI: 0.94-1.00); however, this result was only marginally significant (Table 4). Although the results regarding birth weight were not statistically significant, the effect size was almost analogous to the clinical outcomes in the same direction. Specifically, the odds of an unstable course decreased by 34% with a one-unit increase in neonatal birth weight (OR = 0.66; 95% CI: 0.15-2.89). In addition, the odds of mortality increased by 8% with a one-day increase in the length of hospital stay; however, the results were not significant (OR = 1.08; 95% CI: 0.97-1.20).

**Table 4:** Multivariate logistic regression model showing demographic risk factors associated with the clinical outcome and course of neonatal candidiasis (n = 58) The results of the regression analysis are presented as adjusted odds ratios (ORs) with 95% confidence intervals (CIs). A p-value of <0.05 is considered as statistically significant.

Demographic risk factors associated with clinical outcome							
Risk factors	Correlation coefficient	SE	Wald	p-value	OR	95% CI	
Gestational age	0.08	0.17	0.22	0.64	1.08	0.773	1.52
Age at presentation	0.02	0.10	0.05	0.81	1.02	0.831	1.26
Birth weight	-1.83	0.88	4.32	0.03	0.16	0.023	0.89
Length of hospital stay before candidiasis diagnosis	0.15	0.06	6.15	0.01	1.16	1.03	1.31
Number of antibiotics days	-0.04	0.05	0.73	0.39	0.95	0.86	1.06
Total length of hospital stay	-0.07	0.02	10.58	0.001	0.92	0.88	0.97
Demographic risk factors associated with clinical course							
Gestational age	-0.24	0.16	2.28	0.13	0.78	0.56	1.07
Age at presentation	0.091	0.08	1.081	0.29	1.09	0.92	1.29
Birth weight	-0.41	0.75	0.29	0.58	0.663	0.15	2.89
Length of hospital stay before candidiasis diagnosis	0.08	0.05	2.22	0.13	1.08	0.97	1.20
Number of antibiotics days	-0.034	0.05	0.51	0.47	0.96	0.88	1.06
Total length of hospital stay	-0.03	0.02	3.92	0.04	0.97	0.93	1.00

## Discussion

This study was conducted to estimate the prevalence of neonatal candidiasis, its risk factors, and its adverse outcomes among neonates in a single center in Saudi Arabia. The results showed that almost one-third of neonates included in this study showed *Candida* growth on cultures, with *C. albicans* being the most common causative organism. Regarding the clinical course, 41.4% of the neonates were stable, whereas 58.9% died during the course of treatment. Although our analysis did not reveal any clinically significant risk factors associated with the clinical course and outcome of neonatal candidiasis, it appears that neonates with positive *Candida* blood cultures and disseminated candidiasis were more likely to be unstable and die than those with negative blood cultures and those without disseminated candidiasis, respectively. In addition, neonates who were administered antibiotics before the candidiasis diagnosis had an unstable clinical course compared to neonates who did not receive the same antibiotics. In contrast to clinical factors not being significantly associated with the clinical course and outcomes of neonatal candidiasis, some demographic factors were important risk factors for the clinical course and outcomes of the infection. Neonatal birth weight, length of hospital stay before candidiasis diagnosis, and total length of hospital stay were significant risk factors for mortality in the neonates. High birth weight was a protective factor against mortality and an adverse clinical course of neonatal candidiasis, whereas the extended length of hospital stay before candidiasis diagnosis was a risk factor for mortality and an adverse clinical course of candidiasis. Surprisingly, total length of hospital stay was the only demographic risk factor associated with the clinical course of neonatal candidiasis; however, this association was inverse and statistically non-significant.

The prevalence of *Candida* infection in the present study, which was approximately 30%, is higher than those reported in previous studies [6,8,11,12]. This difference could be due to differences in the methods used to estimate prevalence. Moreover, the differences could be attributed to variations in overcrowding rates, unnecessary use of antibiotics, availability of doctors and nurses, burden on the healthcare system, and the budget spent on healthcare. As in many previous studies, the most common *Candida* species in the present study was *C. albicans* [8,11-17]. Notably, *Candida parapsilosis* was reported to be the predominant *Candida* species in a study by Caparó Ingram et al. [6]. Although *C. albicans* is the most common *Candida* species, other species such as *C. parapsilosis*, *Candida glabrata*, and *Candida tropicalis* have been detected in some studies, indicating that active monitoring of other *Candida* species and the overall epidemiology of *Candida* infection is important [18].

The results of the present study indicate that a prolonged hospital stay beyond seven days is an important risk factor for adverse outcomes of neonatal candidiasis. This is in line with the findings of some previous studies. Caparó Ingram et al. conducted a retrospective paired case-control study and found that neonates with a prolonged hospital stay were 17 times more likely to develop candidiasis than those without a prolonged hospital stay [6]. However, the authors also found that other risk factors, such as the use of umbilical lines, any type of abdominal or thoracic surgery, and treatment with broad-spectrum antibiotics, were associated with neonatal candidemia [6]. Although we did not find these risk factors to be significantly associated with neonatal candidiasis and its outcomes, we found that neonates who were administered antibiotics before the candidiasis diagnosis had an unstable clinical course compared to those who were not. However, this result was not significant and may not have sufficient power for any appreciable difference to be detected. The results of some previous studies have also indicated an association between prolonged hospital stay and an adverse clinical course of neonatal candidiasis [8,12,18,19]. A possible mechanism for this finding is the potential for *Candida* to grow in the hospital environment, which increases further when the neonate is admitted for a prolonged period [18].

We found that birth weight was a protective factor against neonatal candidiasis and its clinical course, which is in turn associated with significant risks of morbidity and mortality. Our findings are comparable to those of other similar studies that have shown that low birth weight is a risk factor for neonatal candidiasis [18,20-23]. Generally, neonates with extremely low birth weights are 10 times more likely to develop neonatal candidiasis than neonates with normal birth weights [24]. This could be because, in contrast to low-birth-weight neonates, neonates with normal birth weight are well-nourished and have grown adequately in utero [25]. Low birth weight may reflect inadequate growth of the baby, as well as a weak immune system, which increases the neonate’s susceptibility to candidiasis and its adverse outcomes [26]. Furthermore, an immature immune system means that low-birth-weight neonates cannot eliminate pathogens from their bodies, thereby making them vulnerable to neonatal candidiasis [27,28].

Strengths and limitations

The main strength of this study is the use of complete medical records, collated between 2010 and 2020, to study the risk factors, etiology, clinical course, and adverse outcomes of neonatal candidiasis. Furthermore, we analyzed the clinical and demographic factors separately and adjusted for important confounders in two different models. In addition, the urine and blood culture tests were performed using standard laboratory measurements and tools.

Despite these strengths, the findings should be interpreted with caution because of the limitations of the study. First, we did not select the neonates randomly; thus, there may be some bias in the results. In addition, the limited number of included neonates may have reduced the power of the study. Second, given that we retrospectively analyzed clinical records rather than following up on the neonates prospectively, there is a possibility of missing some information on important risk factors that were not documented in the records. Third, the observational nature of the study design is limited by the issue of unmeasured confounding factors. However, one may overcome this issue by having an explicit theory about the potential confounders and identifying the confounders using causal diagrams, such as directed acyclic graphs. These graphs can help identify the minimum set of variables that need to be adjusted as potential confounders and that may be highly correlated with the unmeasured confounders. Thus, the problem of unmeasured confounding factors in observational studies can be addressed to a considerable extent. Finally, we did not include all the neonatal units in Saudi Arabia or use a random sample of neonatal units across the country; therefore, the generalizability of the study findings is limited. Future prospective cohort studies with larger sample sizes are required to test the same hypothesis and explore the factors associated with neonatal candidiasis in Saudi Arabia.

## Conclusions

The findings of this study suggest a high prevalence of neonatal candidiasis in Saudi Arabia. In addition, this study showed that the most common risk factors for neonatal candidiasis are prolonged hospital stay, use of antibiotics, and low birth weight. This suggests that additional efforts should be made to screen all neonates admitted to the neonatal intensive care unit for these risk factors and pay special attention to neonates with an increased risk for candidiasis. Although all neonates are fragile, low-birth-weight neonates who have a prolonged hospital stay are more susceptible to candidiasis; thus, resources need to be specifically allocated for the treatment and management of these neonates to reduce the morbidity and mortality rates associated with neonatal candidiasis in Saudi Arabia. The findings of this study can help neonatologists and pediatricians develop a management framework or prophylaxis protocol for neonates susceptible to candidiasis.
